# Main Effect QTL with Dominance Determines Heterosis for Dynamic Plant Height in Upland Cotton

**DOI:** 10.1534/g3.116.034355

**Published:** 2016-08-26

**Authors:** Lianguang Shang, Lingling Ma, Yumei Wang, Ying Su, Xiaocui Wang, Yuhua Li, Abdugheni Abduweli, Shihu Cai, Fang Liu, Kunbo Wang, Jinping Hua

**Affiliations:** * Laboratory of Cotton Genetics, Genomics and Breeding, College of Agronomy and Biotechnology, China Agricultural University, Beijing 100193, China; † Institute of Cash Crops, Hubei Academy of Agricultural Sciences, Wuhan 430064, China; ‡ Institute of Cotton Research, Chinese Academy of Agricultural Sciences/State Key Laboratory of Cotton Biology, Anyang 455000, China

**Keywords:** Upland cotton, plant height, QTL, heterosis, backcross population

## Abstract

Plant height, which shows dynamic development and heterosis, is a major trait affecting plant architecture and has an indirect influence on economic yield related to biological yield in cotton. In the present study, we carried out dynamic analysis for plant height and its heterosis by quantitative trait loci (QTL) mapping at multiple developmental stages using two recombinant inbred lines (RILs) and their backcross progeny. At the single-locus level, 47 QTL were identified at five developmental stages in two hybrids. In backcross populations, QTL identified at an early stage mainly showed partial effects and QTL detected at a later stage mostly displayed overdominance effects. At the two-locus level, we found that main effect QTL played a more important role than epistatic QTL in the expression of heterosis in backcross populations. Therefore, this study implies that the genetic basis of plant height heterosis shows dynamic character and main effect QTL with dominance determines heterosis for plant height in Upland cotton.

Heterosis refers to the phenomenon where a hybrid has a better performance than its parents. The exploitation of heterosis has made a great contribution to agricultural production, and much has been done to explore its genetic basis in order to increase yield. Three classical hypotheses of heterosis have been developed, including dominance, overdominance, and epistasis. The dominance hypothesis argues that the superior performance of the hybrid results from the accumulation of more dominant alleles in the hybrid than in its parents (Jones 1917). The overdominance hypothesis assumes that heterozygosity is responsible for the superior performance over homozygous genotypes (Shull 1908; East 1908). The epistasis hypothesis attributes heterosis to positive epistatic interactions between nonallelic genes (Richey 1942; Yu *et al.* 1997).

The development of molecular markers accelerated our understanding of the genetic basis controlling trait and trait heterosis in crops (Paterson *et al.* 1988). In cotton, some segregation population designs including F_2: 3_, chromosome segment substitution lines (CSSLs), and “immortalized F_2_” (IF_2_) populations were investigated to dissect the genetic basis of yield heterosis (Liu *et al.* 2012; Guo *et al.* 2013; Liang *et al.* 2015). These studies offered different explanations for hybrid vigor in different cotton genetic populations. The quantitative trait loci (QTL) analysis for yield and yield heterosis was studied using the final yield trait at the maturation stage in cotton. These studies ignored the distinct QTL and heterotic QTL actions at different developmental stages.

Plant height is a major trait affecting plant architecture, and it directly determines the biomass and has an indirect influence on economic yield in cotton (Shang *et al.* 2015). In addition, plant height has significant heterosis, and it is a typical trait for studying heterosis (Schnable and Springer 2013). Shen *et al.* (2014) developed a set of 202 CSSLs of an elite rice hybrid to explore the genetic basis of heterosis for plant height at a single locus. The results showed dominance and epistasis to be the main contributors to heterosis for plant height in rice. Wei *et al.* (2015) developed a set of 203 single segment substitution lines (SSSLs) and the testcross population was used to identify heterotic loci for the plant height trait in maize. The results showed that heterosis and trait performance was controlled by different genetic mechanisms, and the single-locus overdominance effect was the main contributor to heterosis for plant height in maize. Li *et al.* (2015) identified a separate QTL for plant height (*qHT7.1*) near the genomic region harboring the known *Dw3* gene using a RIL population. Where two loci have repulsion linkage between two inbreds, heterosis in the hybrid can appear as a single locus with an overdominance mode of inheritance (Lippman and Zamir 2007).

Our recent study of plant height showed that QTL were selectively expressed at different developmental stages and therefore analyzing the genetic basis of quantitative traits only at final maturity in Upland cotton is not representative (Shang *et al.* 2015). In addition, creating “immortalized” backcross populations from recombinant inbred line populations could allow repeated analysis of heterosis (Mei *et al.* 2005). Studies of heterosis using backcross populations were reported in different crops, such as rice (Xiao *et al.* 1995; Li *et al.* 2001, 2008), maize (Frascaroli *et al.* 2007), and rape (Radoev *et al.* 2008). In our previous study, two RIL populations and two corresponding backcross populations were studied to examine the genetic basis of yield and yield heterosis in Upland cotton. The results showed that partial dominance, overdominance, epistasis, and QTL by environment interactions contributed to the yield heterosis in Upland cotton (Shang *et al.* 2016a). However, QTL mapping for quantitative trait at final maturity showed mainly the cumulative effects of QTL. Plant height is a representative dynamic trait but there are no studies reporting on dynamic heterosis QTL for plant height. Therefore, it is important to explore the dynamics of heterotic QTL for plant height at different developmental stages.

In the present study, we used two previously constructed recombinant inbred line populations and their backcross progeny, and conducted QTL analysis for plant height and heterosis performance at five developmental stages in two different environments. The QTL and heterotic QTL were analyzed at the single-locus and two-locus levels. This study will provide new insights into our understanding of the genetic basis of dynamic heterosis in Upland cotton.

## Materials and Methods

### Plant materials

Two hybrids were employed: one called “Xinza 1” (Liang *et al.* 2015; Shang *et al.* 2016b; hereafter referred to as the “XZ hybrid”), derived from a cross of “GX1135” and “GX100-2”; and the other has a common female parent with “Xinza 1,” derived from a cross between “GX1135” and “VGX100-2” (Shang *et al.* 2016a; hereafter referred to as the “XZV hybrid”). “VGX100-2” was selected from “GX100-2” and has significantly different agronomy performance compared with “GX100-2.”

In total, four populations were used based on the experimental design Supplementary Material (Figure S1). The first population was an RIL population. One hundred and seventy-seven RILs of F_10_ generation by single seed descent were developed from an F_1_ individual of the XZ hybrid (Xinza 1). The second population was an RILV population from the XZV hybrid. One hundred and eighty RILs were developed through 10 consecutive selfing generations as well. The third population was a backcross population (BCF_1_) developed from a RIL population of the XZ hybrid. One hundred and seventy-seven BCF_1_ hybrids were developed from a cross where one RIL was used as the female parent and the common parent, GX1135, was used as the male parent, respectively. The fourth population was another backcross population (XZV). One hundred and eighty BCVF_1_ hybrids were developed from crosses between RILs from the RILV population used as the female parent and the common parent, GX1135, used as the male parent, respectively.

In the BC(V)F_1_ population, six-row plots were used which included the BC(V)F_1_ hybrids RIL(V)′ × GX1135 in the middle, and its corresponding female RIL(V)′ and the recurrent parent, GX1135. The trait values for each BC(V)F_1_ hybrid, RIL(V)′ × GX1135, were calculated based on the corresponding female RIL(V)′ and the recurrent parent, GX1135, in each plot to control the error. Each line in the RIL(V)′ population was used as the female parent in the BC(V)F_1_ population and was the same as that in the RIL(V) population. For ease of description, we will refer to the RIL(V)s in the BC(V)F_1_ population as the RIL(V)′ population, respectively. Therefore, in the BC(V)F_1_ population experiments, in both populations 3 (BCF_1_) and 4 (BCVF_1_), each plot consisted of two rows of the female RIL(V)′, BC(V)F_1_ hybrids and GX1135, respectively.

The commercial hybrid of Upland cotton (*Gossypium hirsutum*) was used as a control in our study. This F_1_ hybrid “Ruiza 816” was used as a control (CK) at two locations, Handan (E1) and Cangzhou (E2). It was bred by the Cotton Research Institute of Dezhou (Shandong Province, China) and Biotechnology Research Institute, Chinese Academy of Agricultural Sciences, and was released as a cultivar in Shandong Province and Yellow River Region, China in 2007.

Additionally, two special plots, each consisting of two rows of the XZ hybrid, “Xinza 1” F_1_, and its parents GX1135 and GX100-2, respectively, were used as controls in the population 1 and 3 experiments. Similar controls were set for the population 2 and 4 experiments; each plot consisted of the XZV hybrid F_1_ and its parents GX1135 and VGX100-2.

### Field trials and phenotypic evaluation

The four populations, control, and control hybrids were planted at two locations in 2012: sowing was carried out on April 27, 2012 at the Quzhou Experimental Station of the China Agricultural University, Handan (E1) (36°78′N, 114°92′E), Hebei Province; and on May 4, 2012 at the Guoxin Seed Company Limited, Cangzhou (E2), Hebei Province. Two-row plots were 80 and 50 cm row spacing alternately in E1 and E2. The lengths of plots were 4 m in E1 and 3 m in E2 (Shang *et al.* 2016b). As described above, the hybrid “Ruiza 816” was used as a control (CK) in E1 and E2. In the population 1 and 2 experiments, two-row plots were used with each line; however, in population 3 and 4 experiments, six-row plots were used with each plot consisting of two rows of the BC(V)F_1_ hybrid [RIL(V) × GX1135], and two of each of the corresponding parents: the female RIL(V)′ and GX1135. The field planting followed a randomized complete block design with two replications at each location. The plant density was about 51,000 individuals per hectare in both E1 and E2. Field management followed the local standard field practices.

Plant height was recorded by measuring the main-stem height of individuals and used to map QTL. A total of eight plants, including four consecutive individuals starting from the second plant in both rows in each block, were measured at intervals of 12 d from June 9 to July 27, respectively (*t1*: June 9, *t2*: June 21, *t3*: July 3, *t4*: July 15, and *t5*: July 27).

### DNA isolation and genotype analysis

Young leaves were collected. Extraction of individual genomic DNA and population genotype analysis were carried out following the methods of Liang *et al.* (2013). The PCR conditions were 95° for 3 min, 30 cycles of 95° for 45 s, 55° for 45 s, 72° for 60 s, and 1 cycle at 72° for 10 min. PCR products were run on 8% polyacrylamide gels. A total of 48,836 pairs of SSR primer were used to screen polymorphic loci between three parents. In total, 653 polymorphic loci for the XZ hybrid and 400 for the XZV hybrid were acquired and used to conduct genotype analysis of the populations.

### Data analysis

Basic statistical analysis was implemented using the software SPSS version 19.0 (SPSS, Chicago). The genotype for each BC(V)F_1_ was deduced on the basis of the RIL(V)′s genotype used as the parent for the cross. Correlation analysis between genotype heterozygosity and trait performance in the backcross population was carried out. MAPMAKER 3.0 was used to construct a genetic linkage map using the *Kosambi* mapping function (Lander *et al.* 1987). For XZ and XZV hybrids, QTL analysis was carried out separately for the RIL(V) and BC(V)F_1_ populations. For the RIL(V) and RIL(V)′ populations, the mean trait values from two replications were used as raw data in each location. For each of the BC(V)F_1_ populations, the mean trait values of the BC(V)F_1_s and midparent heterosis (MPH) were used independently as raw data in three locations. Single-locus QTL were conducted using composite interval mapping by WinQTL Cartographer 2.5 in RIL(V)′, RIL(V), BC(V)F_1_, and MPH data (Zeng 1994; Wang *et al.* 2005). A stringent LOD threshold of 2.5 was used to declare suggestive QTL, whereas the same QTL in another environment or population with LOD of at least 2.0 was considered to be a common QTL (Liang *et al.* 2013). The graphic representation of the linkage group and QTL marked was created by Map Chart 2.2 (Voorrips 2002). QTL nomenclature used in rice was employed (McCouch *et al.* 1997). The QTL detected simultaneously in the different data sets allowed an assessment of the degree of dominance (Radoev *et al.* 2008). At the single-locus level, the genetic effects in BC(V)F_1_s were defined as follows: *a* = (P1P1 − P2P2)/2; MPH = *d* = [BC(V)F_1_ − (P1P1 + P2P2)/2] and BC(V)F_1_ = (*a* + *d*) (P1 is the recurrent parent). QTL detected only in co-identified RIL(V) and BC(V)F_1_ and not for MPH were considered as additive. QTL with *d*/*a* ≤ 1 were referred to as being complete or partial dominant loci. QTL with *d*/*a* > 1 or only detectable for MPH data were referred to as overdominant loci. Two-locus analysis that tests the main effect QTL (M-QTL), and digenic epistatic QTL (E-QTL) and their environmental interactions (QTL × environment, QE), was conducted using RIL(V) and BC(V)F_1_ data by the software ICIMapping 4.0 (www.isbreeding.net). A threshold of LOD ≥ 2.5 was used for declaring the presence of M-QTL and LOD ≥ 5.0 E-QTL was used for declaring the presence of E-QTL.

### Data availability

All of our raw data are available as Table S8 and Table S9, which include genotypes and traits of two hybrids. 

## Results

### Phenotypic analysis of plant height

The data for plant height for RIL(V) and BC(V)F_1_ at five stages in XZ and XZV hybrids are shown in Table 1. For XZ and XZV hybrids, the parent GX1135 has higher trait value than GX100-2 and VGX100-2 for plant height at five stages. Apparent heterosis for plant height was observed in the two hybrids. Moreover, the plant height showed different levels of hybrid vigor at different stages. The means of the BC(V)F_1_ population were higher than the RIL(V) population at most stages. The level of heterosis at the final stage was larger than that of early stages. Some extreme lines in the RIL(V) and BC(V)F_1_ populations exceeded hybrids and CK at five stages. Meanwhile, many lines showed higher MPH in the BC(V)F_1_ population than that of the two hybrids (Table 1). An analysis of variance was carried out and significant genotypic variances and environmental variances for plant height were observed at five stages (Table 2). The heritability showed different levels at different developmental stages (Table S1). These results indicated that plant height in populations showed a big variation and this was conducive for QTL analysis.

**Table 1:** Summary statistics on plant height in two hybrids

		Mean			Min			Max			Parents			
*T*	*E*	RIL	BCF_1_	MPH	RIL	BCF_1_	MPH	RIL	BCF_1_	MPH	♀	♂	F_1_	CK
XZ hybrid														
*t*1	*E*1	30.36	30.51	0.13	20.99	23.31	−7.06	41.56	37.75	7.53	31.81	25.97	30.03	35.63
	*E*2	24.62	26.73	−0.31	18.06	20.11	−5.64	30.81	31.81	3.88	26.78	21.19	23.00	29.25
*t*2	*E*1	48.57	48.73	0.60	32.82	38.69	−9.53	67.25	59.94	10.22	52.25	39.88	47.34	55.13
	*E*2	42.47	44.37	0.08	30.88	34.88	−4.88	51.69	52.00	6.44	44.53	35.75	40.34	46.75
*t*3	*E*1	84.69	85.17	1.33	65.30	73.19	−10.13	105.63	99.31	12.28	90.86	70.15	82.66	88.13
	*E*2	76.38	81.85	0.89	57.92	66.31	−6.09	88.06	94.19	9.03	81.16	68.41	76.84	80.13
*t*4	*E*1	103.42	99.73	0.96	79.23	90.44	−7.25	126.25	112.13	10.75	105.88	85.69	95.59	100.72
	*E*2	101.67	109.87	1.10	78.47	87.94	−10.97	116.56	127.38	10.16	107.22	91.48	104.72	105.38
*t*5	*E*1	111.97	105.87	1.11	85.14	96.00	−7.13	134.81	119.81	9.44	110.72	91.27	100.97	104.88
	*E*2	107.25	114.73	1.33	80.31	90.56	−10.56	124.00	135.38	11.16	110.91	93.78	109.91	109.44
XZV hybrid														
*t*1	*E*1	35.94	36.01	0.21	28.81	29.81	−4.00	43.88	42.06	4.84	36.48	30.38	35.16	42.50
	*E*2	29.71	31.77	0.22	23.81	26.38	−3.69	35.69	37.38	5.77	29.78	26.00	29.63	33.69
*t*2	*E*1	53.21	59.29	0.94	42.50	50.94	−5.13	67.00	68.38	7.62	53.47	43.99	50.97	59.00
	*E*2	52.31	55.62	0.56	42.25	47.94	−4.84	61.00	64.81	7.07	52.09	48.13	53.72	55.03
*t*3	*E*1	86.87	90.10	1.44	71.35	77.50	−5.00	108.63	104.69	11.03	89.00	75.33	84.23	90.38
	*E*2	88.40	94.17	1.08	73.25	83.19	−6.53	101.69	104.13	9.66	89.97	79.09	87.34	87.22
*t*4	*E*1	108.08	110.71	1.68	89.60	96.56	−5.19	129.76	122.25	11.00	109.72	93.21	103.16	105.19
	*E*2	110.88	118.96	1.28	93.94	106.45	−10.13	127.25	129.50	10.89	110.34	100.25	114.22	107.63
*t*5	*E*1	114.59	121.04	1.81	93.17	104.13	−6.16	135.81	133.00	9.25	116.09	99.80	111.34	110.75
	*E*2	111.14	120.44	1.80	92.76	106.75	−12.19	127.56	134.88	19.88	112.22	100.59	114.59	110.85

Plant height, measured in centimeters (cm). CK, Ruiza816. Environment: E1, Handan; E2, Cangzhou.

**Table 2:** The results of ANOVA for plant height

Stage	Source of Variation	RIL	BCF_1_	RILV	BCVF_1_
		F	F	F	F
*t*1	*L*	511.41**	233.94**	670.69**	380.68**
	*G*	3.05**	1.98**	1.47**	1.26*
	L*G	1.69**	0.84	1.06	0.68
*t*2	*L*	261.04**	150.99**	6.91**	96.57**
	*G*	3.40**	2.16**	1.73**	0.90
	L*G	1.68**	0.87	1.15	0.66
*t*3	*L*	227.94**	56.13**	6.91**	98.03**
	*G*	2.71**	2.36**	1.43**	1.59**
	L*G	1.21	1.21	1.16	0.87
*t*4	*L*	8.34**	477.91**	21.53**	373.20**
	*G*	2.74**	2.45**	1.92**	1.74**
	L*G	1.20	1.48**	1.03	1.05
*t*5	*L*	47.07**	334.61**	30.63**	1.76**
	*G*	2.47**	2.55**	1.89**	1.48**
	L*G	1.21	1.56**	1.18	0.91

Significance is shown at *P = 0.05, **P = 0.01, respectively. *L*, *G*, and L*G stand for environment, genotype, and environment and genotype interaction effects, respectively.

### Correlations between RIL(V)s and BC(V)F_1_ performance and midparent heterosis

The correlation analyses revealed that different factors determined the backcross hybrid performance (Table 3). Significant high positive correlations between MPH and BC(V)F_1_s performance were observed for all the stages in two environments. It indicated that the levels of heterosis contributed to the variation in the backcross hybrid performance. In addition, all of the trait values of the RIL(V)s and that of their BC(V)F_1_s showed significant positive correlation in two hybrids. This result showed that the variation of the backcross hybrid performance was largely determined by the variation of the mean performance of the RIL(V) parents at five stages. Negative correlations were observed between the RIL(V)s and MPH for all stages.

**Table 3:** Phenotypic correlations between RIL, BCF_1_, and MPH data in two hybrids

Stage	Env.	Between RILs and BCF_1_		Between RILs and MPH		Between BCF_1_ and MPH	
		XZ	XZV	XZ	XZV	XZ	XZV
*t*1	*E*1	0.19*	0.44**	0.00	−0.03	0.66**	0.55**
	*E*2	0.54**	0.39**	0.21**	0.09	0.59**	0.63**
*t*2	*E*1	0.20**	0.51**	−0.08	−0.01	0.65**	0.50**
	*E*2	0.52**	0.23**	0.28**	0.01	0.45**	0.63**
*t*3	*E*1	0.18*	0.51**	−0.18*	0.06	0.58**	0.59**
	*E*2	0.32**	0.23**	0.13	0.01	0.35**	0.62**
*t*4	*E*1	0.28**	0.50**	−0.16*	0.04	0.46**	0.59**
	*E*2	0.34**	0.35**	0.04	−0.02	0.39**	0.58**
*t*5	*E*1	0.33**	0.53**	−0.22**	0.06	0.37**	0.53**
	*E*2	0.32**	0.30**	0.06	−0.04	0.41**	0.63**

Significance is shown at *P = 0.05, **P = 0.01, respectively.

### Relationship between heterozygosity and trait performance

Most of the correlation coefficients are not significant between the heterozygosity of molecular markers and the dynamic performance of BC(V)F_1_ and MPH data for plant height at five stages (Table 4). Overall genome heterozygosity of molecular markers alone had little effect on dynamic plant height performance. Backcross hybrid performance and heterosis might derive from a small amount of genome heterozygosity at not only the early stage but also the final stage. The low correlation coefficients may be attributed to the results of maps with low-density markers and only half of whole-genome heterozygosity existed in backcross populations.

**Table 4:** Correlation between genotypic heterozygosity and dynamic trait performance

Stage	BCF_1_				MPH			
	*E*1		*E*2		*E*1		*E*2	
	XZ	XZV	XZ	XZV	XZ	XZV	XZ	XZV
*t*1	0.15	−0.18*	−0.05	0.00	0.15	0.10	0.04	0.23**
*t*2	0.12	−0.04	−0.11	0.07	0.18*	0.14	0.02	0.13
*t*3	0.03	0.04	−0.15*	0.08	0.12	0.17*	0.00	0.11
*t*4	−0.01	0.11	−0.20**	0.09	0.13	0.13	−0.02	0.11
*t*5	−0.02	0.10	−0.25**	0.03	0.12	0.12	−0.12	0.03

Significance is shown at *P = 0.05, **P = 0.01, respectively.

### Single-locus QTL controlling plant height

Genetic maps for the two hybrid populations were constructed based on the polymorphic loci identified (Figure S2). For the XZ hybrid, 623 loci were mapped to 32 linkage groups and the genetic map spanned 3889.9 cM. For the XZV hybrid, 308 loci were mapped to 39 linkage groups and the genetic map spanned 3048.4 cM (Shang *et al.* 2016a). Single-locus QTL detected for plant height are shown in Table S2 using composite interval mapping. The numbers of different QTL identified by composite interval mapping at five stages in two backcross populations are given in Table 5.

**Table 5:** Effects of QTL identified for plant height by composite interval mapping in two backcross populations

Trait	XZ hybrid				XZV hybrid			
	A	PD	OD	Sum	A	PD	OD	Sum
*t*1	3	3	1	7	1	1	0	2
*t*2	6	3	1	10	0	2	0	2
*t*3	4	2	0	6	1	1	0	2
*t*4	3	1	2	6	2	2	2	6
*t*5	3	0	1	4	3	0	2	5
Total	19	9	5	33	7	6	4	17

A, additive effect; PD, partial dominant effect; OD, overdominant effect.

In the XZ hybrid, a total of 26 QTL were identified. Of 26 QTL, 17 QTL were detected in more than two developmental stages or environments or populations. Interestingly, we observed six QTL for plant height, which were detected at all five stages. In total, 18, 22, 21, 19, and 18 QTL were detected at stages *t*1, *t*2, *t*3, *t*4, and *t*5, respectively. In the backcross population, 19 QTL with an additive effect, nine QTL with a partial dominant effect, and five QTL with an overdominant effect were observed.

In the XZV hybrid, a total of 21 QTL were detected. Of these 21 QTL, 18 were identified in more than two developmental stages or environments or populations. In total, 13, 10, 10, 15, and 14 QTL were identified at stages *t*1, *t*2, *t*3, *t*4, and *t*5, respectively. In the backcross population, seven QTL with an additive effect, six QTL with a partial dominant effect, and four QTL with an overdominant effect were observed.

Conditional QTL mapping was conducted based on adjacent stages from *t–1* to *t* in two hybrid populations. A total of 31 and 24 conditional QTL for plant height were identified in XZ and XZV hybrids, respectively (Table S3). In the XZ hybrid, 24, 16, 16, and 10 conditional QTL were detected at stages ∆*t*1–2, ∆*t*2–3, ∆*t*3–4, and ∆*t*4–5, respectively. In the XZV hybrid, 11, 10, 12, and 10 conditional QTL were detected at stages ∆*t*1–2, ∆*t*2–3, ∆*t*3–4, and ∆*t*4–5, respectively. Two conditional QTL were simultaneously identified during the entire stage of growth. Most of the conditional QTL detected at certain stages showed that the QTL and heterotic QTL were selectively expressed at special stages during plant growth.

### QTL and QE interactions resolved by two-locus analyses

A total of 61 and 49 main effect QTL (M-QTL) and their environmental interactions (QTL × environment interactions, QEs) were respectively detected by inclusive composite interval mapping (ICIM) at five stages of XZ and XZV hybrids (Table 6, Table S4, and Table S5). In the XZ hybrid, a total of 31 and 30 M-QTL and QEs were respectively detected in the RILs and BCF_1_ populations at five developmental stages. On average, the M-QTL explained 4.56 and 3.72% of the phenotypic variation, and the QEs respectively explained 0.56 and 0.86% of the phenotypic variation in the RILs and BCF_1_ populations. In the XZV hybrid, a total of 29 and 20 M-QTL and QEs were respectively detected in the RILVs and BCVF_1_ populations at all developmental stages. On average, the M-QTL explained 2.53 and 2.18% of the phenotypic variation, and the QEs explained 2.70 and 1.97% of the phenotypic variation in the RILVs and BCVF_1_ hybrid data, respectively.

**Table 6:** Summary of M-QTL and E-QTL detected by inclusive composite interval mapping

Stage	RIL			BCF_1_			RILV			BCVF_1_		
M-QTL	*n*	*P*(A)	*P*(AE)	*n*	*P*(A)	*P*(AE)	*n*	*P*(A)	*P*(AE)	*n*	*P*(A)	*P*(AE)
*t*1	9	3.76	1.06	6	5.20	0.77	8	2.35	2.39	2	2.13	2.79
*t*2	4	7.10	0.66	6	6.11	0.34	4	2.12	3.39	3	2.24	3.01
*t*3	6	4.64	0.34	7	3.02	0.77	5	2.47	3.06	3	2.18	1.35
*t*4	6	3.58	0.59	5	2.56	1.10	6	2.87	2.33	5	2.04	1.54
*t*5	6	3.70	0.16	6	1.72	1.30	6	2.84	2.34	7	2.28	1.16
Mean	6.2	4.56	0.56	6.0	3.72	0.86	5.8	2.53	2.70	4.0	2.18	1.97
E-QTL	*n*	*P*(AA)	*P*(AAE)	*n*	*P*(AA)	*P*(AAE)	*n*	*P*(AA)	*P*(AAE)	*n*	*P*(AA)	*P*(AAE)
*t*1	10	2.26	1.76	3	2.78	0.99	2	2.85	1.05	0	0.00	0.00
*t*2	5	2.19	1.72	0	0.00	0.00	1	4.97	0.08	0	0.00	0.00
*t*3	2	2.71	1.76	2	3.02	1.66	0	0.00	0.00	3	5.42	0.25
*t*4	3	2.60	2.69	7	3.24	2.06	0	0.00	0.00	2	3.58	0.97
*t*5	1	3.43	0.96	5	3.56	2.17	2	4.30	0.07	1	5.09	0.00
Mean	4.2	2.64	1.78	3.4	2.52	1.38	1.0	2.42	0.24	1.2	2.82	0.25

*n*, the number of QTL identified. *P*, (in %) was the mean of trait phenotypic variances explained by a single M-QTL or E-QTL.

In total, at the two-locus level, 38 and 11 epistatic QTL (E-QTL) and QEs were respectively detected by ICIM in XZ and XZV hybrids at five developmental stages (Table 6, Table S6, and Table S7). In the XZ hybrid, a total of 21 and 17 E-QTL and QEs were respectively detected in the RILs and BCF_1_ hybrids. On average, the E-QTL explained 2.64 and 2.52% of the phenotypic variation, and the QEs explained 1.78 and 1.38% of the phenotypic variation in the RILs and BCF_1_ hybrid data, respectively. In the XZV hybrid, a total of five and six E-QTL and QEs were detected in the RILVs and BCVF_1_ hybrid data, respectively. On average, the E-QTL explained 2.42 and 2.82% of the phenotypic variation, and the QEs explained 0.24 and 0.25% of the phenotypic variation in the RILVs and BCVF_1_ hybrid data, respectively.

## Discussion

### Dynamic QTL for plant height

Biologically plant height refers to the sum of internode lengths above ground, reflecting the rate of vegetative growth in crops (Shang *et al.* 2015). The incremental values of plant height showed a dynamic development during plant growth in RIL and BC populations. The MPH of the backcross population gradually increases with plant growth, and the performance of heterosis displays a dynamic character. This dynamic performance was conducive for detecting the dynamic heterosis in Upland cotton. Recently, Wang *et al.* (2015) detected a total of 70 QTL for plant height at maturity using a large doubled haploid population containing 348 lines in rapeseed (*Brassica napus*). However, the genetic basis of dynamic heterosis is poorly understood in crop plants. Previous studies involving hQTL mapping provided information about cumulative effects at various stages (Luo *et al.* 2009; Tang *et al.* 2010; Zhou *et al.* 2012; Guo *et al.* 2014). Because they were based on the final value of a quantitative trait, the genetic effects of QTL at the different developmental stages were overlooked. In the present research, different numbers of QTL for plant height were identified at maturity and four other stages of development in two hybrid populations. It was revealed that many of the QTL detected at earlier stages were not detected at final maturity. Our results showed that the QTL and hQTL possessed temporal characteristics (Würschum *et al.* 2014; Shang *et al.* 2015).

### Single-locus dominance contributes to dynamic heterosis for plant height

In the present study, two RIL populations and two corresponding backcross populations were employed to dissect the genetic basis of plant height at single-locus and two-locus levels in Upland cotton. The degree of dominance can be acquired by assessing the QTL detected in RIL(V)s, BC(V)F_1_, and MPH data sets at the single-locus level (Radoev *et al.* 2008). Effects of QTL identified for plant height at various stages are different (Table 5). In the XZV hybrid, a total of one QTL with PD effect and no QTL with OD effect were detected at the *t*1 stage. At the final *t*5 stage, a total of two QTL with PD effect and no QTL with OD effect were detected. These results implied that partial dominance seems more prevalent at an early developmental stage, while the opposite applies at the final maturation stage. In our study, the high level of heterosis for plant height was observed at later stages. The largest number of QTL displayed an overdominance effect at all stages measured. Our results for yield heterosis showed that the QTL for the trait with low heterosis had effects in the partial dominance to full dominance range and the QTL for the trait with high heterosis mainly existed in the full dominance to overdominance range (Shang *et al.* 2016a). There might be more genes involved in later developmental stages and therefore cumulative dominant effects may be more prevalent. The same results were observed in heterotic studies in maize and rapeseed (Frascaroli *et al.* 2007; Radoev *et al.* 2008).

At the two-locus level, some digenic interactions were acquired in two hybrid populations. However, the number of M-QTL and the mean of phenotypic variances explained by M-QTL for plant height are much greater than those for E-QTL at most stages (Table 6). This indicated that single-locus dominance mainly contributed to the performance of hybrid vigor at different developmental stages in Upland cotton. The importance of dominance and overdominance in controlling heterosis at different developmental stages seemed different. Taken together, our results show that dynamic dominance and overdominance contributes to heterosis for the plant height trait during plant growth in Upland cotton.

### Dynamic QTL with epistasis and QE effects

At the two-locus level, some digenic epistatic interactions and QTL × environment interactions were observed in XZ and XZV hybrids (Table 6). The results showed that digenic epistatic interactions and the genotype by environment interactions accounted for a different portion of the phenotypic variation at different developmental stages. QTL with epistasis and QE effects is dynamic at different developmental stages. This phenomenon is in accordance with dynamic growth characteristics for plant height. In the XZ hybrid, the QTL for plant height in backcross populations was more sensitive to the environment than that in RIL populations. Special environmental conditions were essential for hybrid performance in terms of height in backcross populations. More studies that test plant height in different environments are needed (Shang *et al.* 2016a). A previous study in rice also suggested that the QTL × environment interaction effect should be considered in marker assisted selection breeding (Xing *et al.* 2002).

A RIL population was previously used to identify QTL for plant height (Shang *et al.* 2015). In this study, two corresponding backcross populations based on two RIL populations were constructed in order to identify stable QTL. A total of 35 stable QTL were detected in more than one stage, environment, or population. QTL identified from the present study and the QTL from Shang *et al.* (2015) were compared, and 28 common QTL were observed (Table S2). A stable QTL, *qPH-Chr19-1*, which was previously identified was again detected in RIL′, RIL, BCF_1_, and MPH data sets at five developmental stages under two environments, and this QTL could contribute to 5.63–42.66% of the phenotypic variation. These novel stable QTL for plant height detected in multiple stages, populations, and environments will be helpful in fine mapping studies in the future.

## Supplementary Material

Supplemental Material
