# Supplementary material for: Main Effect QTL with Dominance Determines Heterosis for Dynamic Plant Height in Upland Cotton
Source: G3 (Bethesda). 2016 Aug 26;6(10):3373–9. doi: 10.1534/g3.116.034355 (PMC5068956; doi:10.1534/g3.116.034355)
Supplement: Supplemental Material [file supp_6_10_3373__index.html]

Main Effect QTL with Dominance Determines Heterosis for Dynamic Plant Height in Upland Cotton — Supplemental Material 

# Main Effect QTL with Dominance Determines Heterosis for Dynamic Plant Height in Upland Cotton

## Supplemental Material for Shang *et al.*, 2016

**Files in this Data Supplement:**

- Figure S1 - Diagram of genetic populations construction. (.pdf, 66 KB)
- Figure S2 - Locations of QTLs controlling plant height identified in two hybrids. (.pdf, 1 MB)
- Table S1 - The results of broad heritability of plant height. (.pdf, 12 KB)
- Table S2 - QTLs identified for plant height by composite interval mapping in two hybrids. (.pdf, 630 KB)
- Table S3 - Conditional QTLs identified for plant height by composite interval mapping in two hybrids. (.pdf, 704 KB)
- Table S4 - Main effects and environmental interactions detected for plant height in RIL and RILV populations by inclusive composite interval mapping. (.pdf, 127 KB)
- Table S5 - Main effects and environmental interactions detected for plant height in BCF1 and BCVF1 populations by inclusive composite interval mapping. (.pdf, 92 KB)
- Table S6 - Epistatic effects and environmental interactions detected for plant height in RIL and RILV populations using two-locus analysis by inclusive composite interval mapping. (.pdf, 65 KB)
- Table S7 - Epistatic effects and environmental interactions detected for plant height in BCF1 and BCVF1 populations using two-locus analysis by inclusive composite interval mapping. (.pdf, 57 KB)
- Table S8 - Genotypes and plant height trait of XZ hybrid used in this work. (.xls, 2 MB)
- Table S9 - Genotypes and plant height trait of XZV hybrid used in this work. (.xls, 1 MB)
