# Supplementary figures and images for: Main Effect QTL with Dominance Determines Heterosis for Dynamic Plant Height in Upland Cotton
Source: G3 (Bethesda). 2016 Aug 26;6(10):3373–9. doi: 10.1534/g3.116.034355 (PMC5068956; doi:10.1534/g3.116.034355)

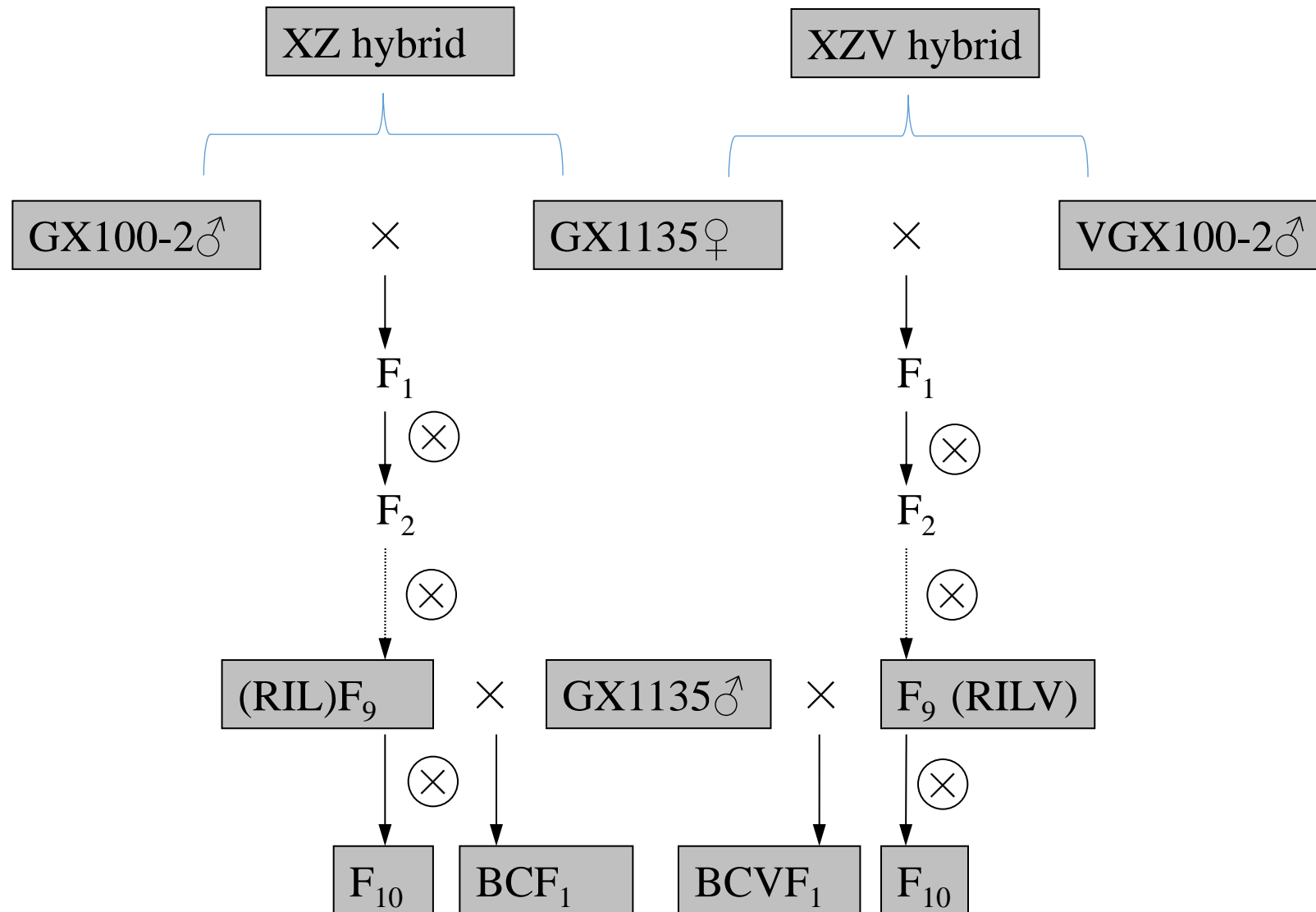

**Figure S1 Diagram of genetic populations construction**

Supplement: Supplemental Material [file supp_g3.116.034355_FigureS1.pdf]
