# Supplementary material for: Main Effect QTL with Dominance Determines Heterosis for Dynamic Plant Height in Upland Cotton
Source: G3 (Bethesda). 2016 Aug 26;6(10):3373–9. doi: 10.1534/g3.116.034355 (PMC5068956; doi:10.1534/g3.116.034355)
Supplement: Supplemental Material [file supp_g3.116.034355_TableS1.pdf]

Table S1 The results of broad heritability of plant height

Stage	RIL (%)	BCF_1 (%)	RILV (%)	$BCVF_1$ (%)
*t*1	44.55	57.40	27.91	45.83
*t*2	50.73	59.82	33.79	27.09
*t*3	55.42	48.74	19.11	45.41
*t*4	56.16	39.46	46.15	39.39
*t*5	50.91	38.75	37.67	38.41
