# Supplementary material for: Main Effect QTL with Dominance Determines Heterosis for Dynamic Plant Height in Upland Cotton
Source: G3 (Bethesda). 2016 Aug 26;6(10):3373–9. doi: 10.1534/g3.116.034355 (PMC5068956; doi:10.1534/g3.116.034355)
Supplement: Supplemental Material [file supp_g3.116.034355_TableS2.pdf]

Table S~~1~~2 QTLs identified for plant height by composite interval mapping in two hybrids

QTL	T	Env.	Flanking markers		RIL′			RIL			BC			MPH			d/a	Type
					LOD	A	Var (%)	LOD	A	Var (%)	LOD	A+D	Var (%)	LOD	D	Var (%)		
XZ hybrid																		
qPH-Chr2-2	*t*2	E1	PGML0700	SWU12016										3.03	-1.1	10.46		OD
qPH-Chr4-1	*t*2	E2	NAU2701	SWU18876							3.39	0.87	5.32					A
	*t*3	E1	SWU18881	NAU2701	2.82	1.31	3.86											
	*t*4	E1	SWU18881	NAU2701	2.67	1.34	4.18											
	*t*4	E2	NAU2701	SWU18876							3.48	1.98	7.29					A
	*t*5	E2	NAU2701	SWU18876							3.47	2.18	7.25					A
qPH-Chr5-1	*t*1	E1	PGML1917	SWU17715	3.71	0.85	7.02	5.32	2.32	26.87								
	*t*2	E1	PGML1917	SWU17715	5.83	1.98	14.46	5.59	3.05	20.49								
	*t*3	E1	PGML1917	SWU17715	9.7	2.98	20.17	8.49	4.01	24.63								
	*t*3	E1	PGML1917	SWU17715														
	*t*4	E2	PGML1917	SWU17715				3.02	3.19	18.21								
	*t*5	E1	PGML1917	SWU17715	10.65	3.64	26.41											
	*t*5	E2	PGML1917	SWU17715	2.85	3.48	15.23											
qPH-Chr5-2	*t*1	E1	TMB1296	HAU1603				5.59	1.39	9.64								
	*t*2	E1	TMB1296	HAU1603	7.22	1.63	9.74	5.81	2.11	9.81								
	*t*3	E1	TMB1296	HAU1603	9.79	2.58	14.97	8.06	3.10	14.63								
	*t*4	E1	TMB1296	HAU1603	10.41	2.78	18.39	6.78	3.12	13.05								
	*t*4	E2	HAU1603	PGML4457				3.28	1.83	5.94								
	*t*5	E1	TMB1296	HAU1603	11.89	3.31	21.44	8.58	3.73	16.69								

QTL	T	Env.	Flanking markers		RIL′			RIL			BC			MPH			d/a	Type
					LOD	A	Var (%)	LOD	A	Var (%)	LOD	A+D	Var (%)	LOD	D	Var (%)		
	*t*5	E2	HAU1603	PGML4457				3.09	2.04	5.69								
qPH-Chr5-3	*t*2	E1	PGML4350	SWU17781	3.92	1.3	6.32											
qPH-Chr5-4	*t*5	E1	SWU17787	SWU13378							3.41	1.42	9.24					A
qPH-Chr6-1	*t*3	E2	ICR02737	TMB2940	3.71	1.81	6.62											
qPH-Chr7-1	*t*1	E1	SWU10205	HAU1483a							3.86	-0.88	8.10					A
	*t*2	E2	SWU10205	HAU1483a							2.52	-0.75	3.94					A
	*t*3	E2	SWU10205	HAU1483a							3.22	-1.49	6.21					A
qPH-Chr10-1	*t*3	E2	SWU20260	Gh144				2.94	-1.48	5.14								
	*t*4	E2	SWU20260	Gh144				5.18	-2.39	9.65								
	*t*5	E2	SWU20260	Gh144				3.75	-2.29	6.99								
qPH-Chr10-2	*t*2	E1	HAU0635	NAU2139				3.2	-1.58	5.42								
	*t*4	E2	Gh320	HAU0635				3.25	-2.35	9.73								
	*t*5	E1	Gh320	HAU0635				3.09	-2.51	7.54								
qPH-Chr11-1	*t*4	E2	SWU16281	SWU16269				2.55	1.64	4.82								
	*t*5	E2	SWU12282	Gh316	3.24	2.37	6.60											
	*t*5	E2	SWU16281	SWU16269				3.33	2.16	6.39								
qPH-Chr13-1	*t*1	E2	SWU22413	CGR5331							2.53	-0.55	4.99				2.67	OD
	*t*1	E2	CGR5554	SHIN1462										2.99	-0.4	6.12		
qPH-Chr13-2	*t*3	E1	NAU3468	SWU22309	3.71	-1.63	5.82											
qPH-Chr13-3	*t*2	E1	SWU22309	SWU22324	3.69	-1.32	6.02											
qPH-Chr14-1	*t*5	E1	CGR6784	NAU874	2.51	-1.46	3.98											
qPH-Chr16-1	*t*1	E2	SWU10214	Gh56	3.38	0.65	5.4											

QTL	T	Env.	Flanking markers		RIL′			RIL			BC			MPH			d/a	Type
					LOD	A	Var (%)	LOD	A	Var (%)	LOD	A+D	Var (%)	LOD	D	Var (%)		
qPH-Chr19-1	*t*1	E1	Gh616	CIR139	12.53	1.53	22.03				6.84	1.11	12.66		(±0.42)		0.38	PD
	*t*1	E1	Gh616	CIR139				6.58	1.53	11.43								
	*t*1	E2	Gh616	CIR139	21.62	1.86	42.66											
	*t*1	E2	NAU5330	Gh72				20.26	1.63	35.02								
	*t*1	E2	Gh616	CIR139							18.38	1.41	32.09	4.43	0.49	9.29	0.3	PD
	*t*2	E1	NAU5330	Gh72	18.15	2.83	29.1											
	*t*2	E1	Gh616	CIR139				9	2.69	15.7	10.03	2.01	19.59		(±0.68)		0.25	PD
	*t*2	E2	Gh616	CIR139	18.59	2.76	35.26											
	*t*2	E2	NAU5330	Gh72				18.9	2.65	34.55								
	*t*2	E2	Gh616	CIR139							12.83	1.89	23.81	4.01	0.57	8.36	0.43	PD
	*t*3	E1	NAU5330	Gh72	12.25	3.07	21.09											
	*t*3	E1	Gh616	CIR139				4.95	2.37	8.46	7.38	1.98	14.1		(±0.39)		0.16	PD
	*t*3	E2	Gh616	CIR139	8.51	2.91	17.06											
	*t*3	E2	Gh616	CIR139				11.43	3.09	22.18								
	*t*3	E2	DC40122	NAU833a							4.01	1.74	7.78		(±1.35)		0.44	PD
	*t*4	E1	NAU5330	Gh72	4.72	1.82	7.81											
	*t*4	E1	Gh616	CIR139							5.17	1.49	10.28		(±0.33)		0.18	PD
	*t*4	E2	Gh616	CIR139	3.11	2.2	6.66											
	*t*5	E1	NAU5330	Gh72	3.94	1.87	6.59											
	*t*5	E2	NAU5330	Gh72				3.06	2.03	5.63								
qPH-Chr19-2	*t*1	E1	NAU1042	NAU3437	9.26	1.34	16.95				4.18	0.87	7.99		(±0.47)		0.35	PD
	*t*1	E2	NAU1042	NAU3437				13.44	1.49	29.23								

QTL	T	Env.	Flanking markers		RIL′			RIL			BC			MPH			d/a	Type
					LOD	A	Var (%)	LOD	A	Var (%)	LOD	A+D	Var (%)	LOD	D	Var (%)		
	*t*2	E1	NAU1269	NAU828	12.45	2.45	21.2											
	*t*2	E1	NAU1042	NAU3437										2.69	0.86	6.25	0.32	PD
	*t*2	E2	NAU1042	NAU3437				13.8	2.7	36.07	8.91	1.81	22.73					
	*t*3	E2	NAU1042	NAU3437				9.19	3.36	26.95								
	*t*4	E2	NAU833a	NAU1269				3.78	2.02	6.95								
qPH-Chr20-1	*t*1	E1	CGR6154	SWU20246							3.18	-1.17	14.79					A
	*t*2	E1	CGR6154	SWU20246	3.39	-1.09	4.33											
	*t*3	E1	CGR6154	SWU20246	2.63	-1.74	6.88											
	*t*4	E1	CGR6154	SWU20246							2.68	-1.79	15.38					A
	*t*5	E1	CGR6154	SWU20246							4.37	-2.23	22.78					
qPH-Chr20-2	*t*2	E1	CER0167	SWU20064							2.74	-1.38	9.52					A
	*t*2	E2	CER0167	SWU20064							3.14	-1.15	9.52					A
	*t*3	E1	CER0167	SWU20064							2.96	-1.69	10.69					A
	*t*3	E2	CER0167	SWU20064	3.03	-2.12	9.61											
	*t*4	E2	CER0167	SWU20064							2.50	-2.32	9.86					A
qPH-Chr20-3	*t*1	E1	DPL0319	HAU1378				5.26	1.4	9.83								
	*t*2	E1	SWU20035	DPL0319				5.57	2.37	12.05								
	*t*3	E1	SWU20035	DPL0319				2.71	2.19	7.26								
	*t*4	E1	SWU20035	DPL0319				2.83	2.44	8.01								
	*t*5	E1	SWU20035	DPL0319				3.73	2.83	9.40								
qPH-Chr21-1	*t*1	E1	DPL0777	CGR5217							3.94	0.85	7.62					A
	*t*2	E1	DPL0777	CGR5217							3.71	1.20	6.97					A

QTL	T	Env.	Flanking markers		RIL′			RIL			BC			MPH			d/a	Type
					LOD	A	Var (%)	LOD	A	Var (%)	LOD	A+D	Var (%)	LOD	D	Var (%)		
	*t*3	E1	DPL0777	CGR5217							3.66	1.4	7.16					A
	*t*4	E1	CGR5217	BNL3442a					(±0.51)		4.29	1.34	8.44				1.63	OD
	*t*4	E1	CGR5217	BNL3442a										2.84	0.83	5.93		
	*t*5	E1	DPL0777	CGR5217							4.42	1.40	8.93					A
qPH-Chr24-1	*t*5	E2	BNL1521	HAU2504				3.67	2.64	9.52								
qPH-Chr25-1	*t*3	E1	Gh220	SWU19434				2.54	1.94	5.63								
	*t*4	E1	Gh220	SWU19434				2.60	2.17	6.22								
qPH-Chr26-1	*t*2	E1	CER0144	CGR5452							4.03	-1.18	6.95					A
	*t*3	E1	CGR6930	SWU17241							3.78	-1.35	6.88					A
	*t*4	E1	CER0144	CGR5452										4.18	-1.02	8.86		OD
	*t*5	E1	CER0144	CGR5452										3.19	-0.86	6.74		OD
qPH-Chr26-2	*t*1	E2	SWU16753	SWU16780	2.81	-0.56	3.99											
	*t*1	E2	SWU16753	SWU16780				3.33	-0.58	4.39								
XZV hybrid																		
qPH-Chr1-1	*t*1	E1	HAU2489	DPL0790				4.53	0.80	7.31								
	*t*2	E1	HAU2489	DPL0790				2.60	0.88	4.08								
	*t*5	E1	HAU2489	DPL0790				2.63	1.42	3.42								
qPH-Chr4-1	*t*1	E1	SWU16783	SWU18876	3.65	0.82	9.80				4.07	0.94	18.56		(±0.12)		0.15	PD
	*t*2	E1	SWU16783	SWU18876	3.02	1.03	6.49				3.12	1.14	12.2		(±0.11)		0.11	PD
qPH-Chr6-1	*t*3	E2	BNL3650	TMB2940	2.63	1.48	8.83											
	*t*5	E2	BNL3650	TMB2940	3.59	2.09	8.65											
qPH-Chr6-2	*t*1	E2	HAU0921	NAU2768	3.07	0.64	6.62											

QTL	T	Env.	Flanking markers		RIL′			RIL			BC			MPH			d/a	Type
					LOD	A	Var (%)	LOD	A	Var (%)	LOD	A+D	Var (%)	LOD	D	Var (%)		
	*t*5	E2	HAU0921	NAU2768	2.51	1.64	5.25											
qPH-Chr9-1	*t*1	E1	SHIN0830	Gh111	4.10	0.71	7.36											
	*t*3	E2	SHIN0830	Gh111				3.63	1.53	7.43								
qPH-Chr13-1	*t*2	E1	SWU13032	HAU2850				3.42	-1.23	8.15								
	*t*3	E1	SWU13032	HAU2850				3.73	-2.17	9.94								
	*t*4	E1	SWU13032	HAU2850				4.55	-2.29	9.48								
	*t*4	E2	SWU13032	HAU2850	3.08	-1.90	8.90	3.08	-1.97	9.01	3.12	-1.46	10.02		(±0.51)		0.26	PD
qPH-Chr13-2	*t*2	E1	NAU3398	CGR5331				3.63	-1.04	5.76								
	*t*3	E1	NAU3398	CGR5331				3.55	-1.62	5.46								
	*t*4	E1	NAU3398	CGR5331				5.02	-1.98	7.09								
	*t*4	E2	NAU3398	CGR5331	3.19	-1.59	6.19											
	*t*5	E1	NAU3398	CGR5331				5.28	-2.03	7.10								
	*t*5	E2	NAU3398	CGR5331	3.44	-1.83	7.01											
qPH-Chr14-1	*t*1	E1	DPL0502	ICR00401	3.05	0.63	5.95											
qPH-Chr14-2	*t*1	E2	TMB0071	HAU1000				2.69	-0.49	4.94								
	*t*2	E2	TMB0071	HAU1000	4.20	-1.02	8.72	4.91	-1.10	9.87								
	*t*3	E2	TMB0071	HAU1000	2.72	-1.24	6.17											
	*t*4	E2	TMB0071	HAU1000	3.60	-1.78	7.73											
	*t*4	E2	HAU1000	TMB1931				3.27	-1.69	6.46								
qPH-Chr15-1	*t*3	E2	NAU3736	SWU11691	3.42	1.54	9.52											
qPH-Chr16-1	*t*1	E2	SWU10211	SWU10266				3.22	0.53	5.85								
	*t*4	E2	HAU1129	C2_0011B				4.08	2.35	12.84								

QTL	T	Env.	Flanking markers		RIL′			RIL			BC			MPH			d/a	Type
					LOD	A	Var (%)	LOD	A	Var (%)	LOD	A+D	Var (%)	LOD	D	Var (%)		
	*t*5	E2	HAU1129	C2_0011B				4.14	2.77	15.16								
qPH-Chr16-2	*t*2	E2	SWU10211	SWU10266				3.6	0.92	7.06								
	*t*4	E2	SWU10211	SWU10266				4.9	2.06	9.74								
	*t*5	E2	SWU10211	SWU10266				4.63	2.23	9.73								
qPH-Chr16-3	*t*4	E2	SWU10266	SWU18366				3.04	2.55	14.99								
qPH-Chr18-1	*t*1	E2	TMB1638	CGR6812	2.89	-0.79	10.15	4.29	-0.86	15.55								
	*t*2	E2	TMB1638	CGR6812	2.77	-0.98	8.12	3.72	-1.38	15.69								
qPH-Chr21-1	*t*4	E1	JESPR154	BNL5602							2.75	-1.12	5.61					A
	*t*5	E1	JESPR154	BNL5602							3.63	-1.61	7.98					A
qPH-Chr23-1	*t*1	E1	CGR5158	HAU1758				4.96	-1.52	25.69								
	*t*2	E1	CGR5158	HAU1758				4.42	-1.26	7.6								
	*t*3	E1	CGR5158	HAU1758				8.72	-2.8	15.02								
	*t*4	E1	CGR5158	HAU1758				8.3	-2.76	12.59								
	*t*5	E1	CGR5158	HAU1758				8.67	-2.86	12.58								
qPH-Chr23-2	*t*4	E2	BNL3482	HAU0244										3.65	-1.31	11.11		OD
	*t*5	E2	BNL3482	HAU0244										2.53	-1.27	6.41		OD
qPH-Chr23-3	*t*1	E1	NAU2140	DC40286	3.24	-0.67	6.84											
	*t*2	E1	NAU2140	DC40286	5.05	-1.31	10.93				6.77	-1.4	18.14		(±0.09)		0.07	PD
	*t*3	E2	NAU2140	DC40286				3.49	1.76	10.02								
	*t*5	E1	NAU2140	DC40286							4.43	-1.91	11.09					A
qPH-Chr23-4	*t*1	E1	DC40286	PGML1434				7.76	-1.16	15.51	4.92	-0.88	13.07					
	*t*1	E2	DC40286	PGML1434				5.55	0.74	11.07								

QTL	T	Env.	Flanking markers		RIL′			RIL			BC			MPH			d/a	Type
					LOD	A	Var (%)	LOD	A	Var (%)	LOD	A+D	Var (%)	LOD	D	Var (%)		
	*t*2	E1	DC40286	PGML1434				8.60	-1.71	15.41								
	*t*3	E1	DC40286	PGML1434	3.22	-1.43	7.33	7.86	-2.56	13.52	3.97	-1.35	9.2		(±1.21)		0.47	PD
	*t*4	E1	DC40286	PGML1434				11.44	-3.2	18.29	3.06	-1.25	6.83		(±1.95)		0.61	PD
	*t*5	E1	DC40286	PGML1434				14.57	-4.23	23.25								
qPH-Chr26-1	*t*1	E2	CGR6477	PGML2562							3.46	-0.55	9.15					A
	*t*3	E2	CGR6477	PGML2562							2.56	-1.34	11.51					A
	*t*4	E2	CGR6477	PGML2562							3.17	-1.56	11.38					A
	*t*5	E2	HAU1571	CGR6477							3.07	-1.46	8.57					A
qPH-Chr26-2	*t*4	E2	SWU16721	SWU16680										3.27	1.01	7.31		OD
	*t*5	E2	SWU16721	SWU16680										3.24	1.31	7.46		OD

Bold figures indicate the QTL was detected in more than two environments or populations simultaneously

QTL identified from present study and the QTL from Shang et al.(2015) were compared, and common QTLs are shown as blue figures

Env., Environment, E1: Handan; E2: Cangzhou; T, Stage t1, t2, t3, t4, t5

Effect, the genetic expectation of a QTL effect obtained is the additive effect (A) when estimated from the RILs and RIL′s, the additive and dominance effects (A+D) from the BCF_1 mean values

Var%, Phenotypic variation explained by a single QTL

d/a, dominance ratio

Type, The QTLs which were identified in backcross population were grouped into three types: (A) additive effect, (PD) partial dominance effect, (OD) overdominance effect.
