# Supplementary material for: Main Effect QTL with Dominance Determines Heterosis for Dynamic Plant Height in Upland Cotton
Source: G3 (Bethesda). 2016 Aug 26;6(10):3373–9. doi: 10.1534/g3.116.034355 (PMC5068956; doi:10.1534/g3.116.034355)
Supplement: Supplemental Material [file supp_g3.116.034355_TableS3.pdf]

Table S3 Conditional QTLs identified for plant height by composite interval mapping in two hybrids

QTL	Stage	Env.	Flanking markers		RIL′			RIL			BC			MPH		
					LOD	A	Var%	LOD	A	Var%	LOD	A+D	Var%	LOD	D	Var%
XZ hybrid																
qPH-Chr1-1	△*t*4-5	E2	ICR03724	ICR03725	3.96	0.52	8.41									
	△*t*4-5	E2	ICR03725	SWU10987	3.40	0.49	7.48									
qPH-Chr1-2	△*t*2-3	E2	SWU10994	HAU1001				3.34	-0.71	6.66						
qPH-Chr2-1	△*t*4-5	E1	SWU12025	SWU11889										3.19	0.42	10.03
qPH-Chr2-2	△*t*1-2	E1	SHIN0219	TMB2386										5.22	-0.77	20.71
qPH-Chr2-3	△*t*1-2	E1	SWU14060	NBRI0014										3.34	0.43	7.08
qPH-Chr5-1	△*t*4-5	E2	SWU20913	Gh260				4.00	0.58	8.13						
qPH-Chr5-2	△*t*1-2	E1	PGML1917	SWU17715	7.05	1.02	17.16									
	△*t*1-2	E1	PGML1917	SWU17715				3.39	0.72	6.69						
	△*t*2-3	E1	PGML1917	SWU17715				6.30	1.58	25.44						
	△*t*2-3	E1	SWU17715	Gh388	7.72	1.02	15.46									
	△*t*3-4	E2	PGML1917	SWU17715	4.70	2.05	30.88									
	△*t*4-5	E2	PGML1917	SWU17715	3.33	0.75	17.96									
qPH-Chr5-3	△*t*1-2	E1	TMB1296	HAU1603	7.14	0.77	9.73									
	△*t*3-4	E2	TMB1296	HAU1603	2.70	0.86	5.42									
	△*t*2-3	E1	HAU1603	PGML4457							2.72	0.45	5.54			
	△*t*2-3	E1	HAU1603	PGML4457				5.31	1.02	10.28						
qPH-Chr5-4	△*t*2-3	E2	MUSS193	PGML4350				3.35	0.71	6.56						
	△*t*1-2	E1	PGML4350	SWU17781	3.88	0.62	6.29									

QTL	Stage	Env.	Flanking markers		RIL′			RIL			BC			MPH		
					LOD	A	Var%	LOD	A	Var%	LOD	A+D	Var%	LOD	D	Var%
qPH-Chr6-1	Δt2-3	E2	BNL3650	ICR10602	4.12	0.97	9.41									
qPH-Chr6-2	Δt2-3	E2	ICR02737	TMB2940	4.22	0.96	8.92									
qPH-Chr10-1	Δt2-3	E2	SWU20260	Gh144				2.93	-0.67	5.74						
	Δt3-4	E2	SWU20260	Gh144				3.18	-0.81	5.97						
qPH-Chr11-1	Δt2-3	E1	ICR01787	NAU3695										3.20	0.49	7.19
qPH-Chr11-2	Δt2-3	E1	SWU15972	TMB0628				2.99	1.02	10.67						
	Δt3-4	E2	TMB0628	HAU0639	3.89	1.02	7.64									
qPH-Chr12-1	Δt4-5	E1	DPL0252	BNL3261							4.14	0.45	10.72			
qPH-Chr13-1	Δt1-2	E1	NAU3468	SWU22309	4.38	-0.63	6.31									
	Δt1-2	E2	SWU22309	SWU22324				4.60	-0.77	10.82						
qPH-Chr14-1	Δt3-4	E2	NAU874	SWU13824				3.25	-0.82	6.12						
	Δt4-5	E1	NAU874	SWU13824							5.15	-0.49	12.06			
	Δt4-5	E1	CGR6683	NAU3308	3.08	-0.40	6.11									
qPH-Chr19-1	Δt1-2	E2	NAU5330	Gh72				9.50	1.04	20.16						
	Δt1-2	E1	NAU5330	Gh72	18.86	1.38	30.57									
	Δt1-2	E1	Gh616	CIR139							9.04	0.82	17.51			
	Δt1-2	E1	Gh616	CIR139				8.27	1.09	14.89						
	Δt1-2	E2	Gh616	CIR139							4.97	0.76	11.35			
	Δt1-2	E2	Gh616	CIR139	9.75	1.04	20.71									
	Δt3-4	E1	Gh616	CIR139							4.62	-0.72	9.11			
	Δt3-4	E1	Gh616	CIR139	9.82	-1.26	19.28									
qPH-Chr19-2	Δt1-2	E1	NAU1269	NAU828	15.20	1.27	25.62									

QTL	Stage	Env.	Flanking markers		RIL′			RIL			BC			MPH		
					LOD	A	Var%	LOD	A	Var%	LOD	A+D	Var%	LOD	D	Var%
	Δ*t*1-2	E1	NAU1042	NAU3437							7.78	0.88	19.99			
	Δ*t*1-2	E2	NAU1042	NAU3437	6.47	0.99	18.87									
	Δ*t*1-2	E2	NAU1042	NAU3437							3.84	0.82	14.07			
	Δ*t*3-4	E1	NAU1042	NAU3437							4.73	-0.92	15.16			
	Δ*t*3-4	E1	NAU1042	NAU3437	6.18	-1.22	19.17									
qPH-Chr19-4	Δ*t*2-3	E1	SWU14431b	SWU17782							4.49	0.86	15.63			
qPH-Chr20-1	Δ*t*1-2	E2	CER0167	SWU20064	2.54	-0.71	10.05									
	Δ*t*3-4	E1	CER0167	SWU20064							3.68	0.86	13.45			
	Δ*t*3-4	E1	CER0167	SWU20064										3.45	0.67	9.85
qPH-Chr20-2	Δ*t*3-4	E1	SWU20035	DPL0319										2.92	0.54	6.40
	Δ*t*3-4	E1	SWU20035	DPL0319							4.19	0.78	10.95			
qPH-Chr21-1	Δ*t*3-4	E2	SWU0189	DPL0050a				4.53	-1.01	9.46						
qPH-Chr21-2	Δ*t*4-5	E2	CGR5808	HAU0423	4.47	0.54	9.17									
qPH-Chr23-1	Δ*t*3-4	E1	Gh327	SWU14770	3.13	0.65	5.62									
qPH-Chr24-1	Δ*t*1-2	E2	BNL1521	HAU2504				3.24	0.62	7.34						
	Δ*t*4-5	E2	BNL1521	HAU2504				2.94	0.55	7.21						
qPH-Chr25-1	Δ*t*1-2	E1	HAU1382	SWU19848				2.81	0.60	4.71						
	Δ*t*1-2	E2	HAU1382	SWU19848	2.64	0.51	4.80									
	Δ*t*2-3	E2	HAU1382	SWU19848	2.57	0.72	5.25									
	Δ*t*1-2	E2	SWU19848	CGR6864				2.70	0.59	6.74						
qPH-Chr26-1	Δ*t*2-3	E1	HAU1571	BNL598	3.07	0.62	5.67									
qPH-Chr26-2	Δ*t*2-3	E1	BNL598	PGML1637	3.30	0.69	7.23									

QTL	Stage	Env.	Flanking markers		RIL′			RIL			BC			MPH		
					LOD	A	Var%	LOD	A	Var%	LOD	A+D	Var%	LOD	D	Var%
qPH-Chr26-3	∆*t*1-2	E1	CGR6930	SWU17241							3.36	-0.48	5.99			
	∆*t*2-3	E2	CGR6930	SWU17241										3.06	-0.43	6.94
qPH-Chr26-4	∆*t*3-4	E1	C2_0135	PGML2321	3.03	0.75	7.49									
XZV hybrid																
qPH-Chr1-1	∆*t*4-5	E2	ICR03725	ICR03724	3.34	0.86	8.03									
qPH-Chr2-1	∆*t*2-3	E2	SWU11889	JESPR304				3.05	0.95	6.57						
	∆*t*2-3	E1	TMB1268	SWU11976							2.63	-0.63	5.51			
qPH-Chr11-1	∆*t*1-2	E1	CGR5421	MUSS278	2.65	-0.55	5.62									
	∆*t*1-2	E1	CGR5421	MUSS278				2.65	-0.57	5.26						
qPH-Chr12-1	∆*t*1-2	E2	Gh568	CGR5111	4.14	0.77	8.52									
qPH-Chr13-1	∆*t*1-2	E1	SWU13032	HAU2850				3.89	-0.79	10.42						
	∆*t*1-2	E1	SWU13032	HAU2850	3.97	-0.72	9.54									
qPH-Chr13-2	∆*t*1-2	E1	NAU3398	CGR5331				4.53	-0.81	10.73						
qPH-Chr14-1	∆*t*4-5	E1	HAU1000	TMB1931				7.98	0.75	19.09						
qPH-Chr14-2	∆*t*1-2	E2	BNL3661	PGML4891	3.10	0.66	6.29									
	∆*t*1-2	E2	BNL3661	PGML4891							3.25	0.59	6.78			
qPH-Chr15-1	∆*t*2-3	E2	NAU3736	SWU11691	3.01	0.81	8.66									
qPH-Chr16-1	∆*t*2-3	E1	DPL0897	SWU20341							3.43	0.72	7.13			
qPH-Chr20-1	∆*t*3-4	E1	SWU20246	Gh451							3.40	-0.69	7.15			
	∆*t*3-4	E1	SWU20246	Gh451					(±0.11)					3.80	-0.58	8.27
qPH-Chr20-2	∆*t*2-3	E1	DPL0131	DPL0777							3.84	-0.76	8.02			
	∆*t*2-3	E1	DPL0131	DPL0777				2.60	-0.79	5.05						

QTL	Stage	Env.	Flanking markers		RIL′			RIL			BC			MPH		
					LOD	A	Var%	LOD	A	Var%	LOD	A+D	Var%	LOD	D	Var%
	△*t*2-3	E1	DPL0131	DPL0777	4.00	-0.99	10.80									
qPH-Chr20-3	△*t*2-3	E1	DPL0777	HAU0423							2.67	-0.72	7.30			
	△*t*3-4	E2	DPL0777	HAU0423	3.17	-1.14	10.16									
qPH-Chr20-4	△*t*4-5	E1	HAU0423	JESPR154	3.86	-0.74	11.48									
	△*t*3-4	E2	HAU0423	JESPR154				3.18	-1.45	9.73						
qPH-Chr20-5	△*t*3-4	E2	BNL5602	JESPR251				3.22	-1.21	6.78						
qPH-Chr21-1	△*t*3-4	E1	SWU16370	CGR5748				3.26	-0.60	6.41						
qPH-Chr21-2	△*t*3-4	E1	CGR5748	PGML2500				3.10	-0.64	7.41						
qPH-Chr23-1	△*t*2-3	E1	CGR5158	HAU1758				5.79	-1.21	10.98						
qPH-Chr23-2	△*t*1-2	E1	NAU2140	DC40286				3.00	-0.70	7.82						
	△*t*4-5	E2	NAU2140	DC40286	3.70	-1.04	11.99									
qPH-Chr23-3	△*t*1-2	E1	DC40286	PGML1434							6.89	-0.66	14.41			
	△*t*1-2	E1	DC40286	PGML1434	5.12	-0.82	12.32									
	△*t*2-3	E1	DC40286	PGML1434				3.55	-0.95	6.94						
	△*t*3-4	E1	DC40286	PGML1434				3.38	-0.72	8.83						
	△*t*4-5	E2	DC40286	PGML1434							4.66	-0.81	10.66			
	△*t*4-5	E2	DC40286	PGML1434	4.32	-1.10	13.24									
	△*t*4-5	E1	DC40286	PGML1434							4.16	-0.65	11.10			
qPH-Chr23-4	△*t*4-5	E1	PGML1434	MUSB994							3.76	-0.85	19.34			
	△*t*3-4	E1	PGML1434	MUSB994				3.21	-1.02	18.70						
qPH-Chr23-5	△*t*4-5	E1	NAU3588	NAU5373a	3.79	0.61	7.95									
qPH-Chr25-1	△*t*3-4	E1	NAU2968	DPL0377	5.89	-1.29	14.37									

QTL	Stage	Env.	Flanking markers		RIL′			RIL			BC			MPH		
					LOD	A	Var%	LOD	A	Var%	LOD	A+D	Var%	LOD	D	Var%
	△*t*3-4	E2	DPL0377	SWU19413							4.44	-0.86	11.22			
qPH-Chr26-1	△*t*3-4	E1	SWU16777	SWU16780				3.03	-0.59	5.96						
	△*t*4-5	E2	SWU16780	SWU16735							3.20	0.64	6.75			

Bold figures indicate the QTL was detected in more than two environments or populations simultaneously

Env., Environment, E1: Handan; E2: Cangzhou

Effect, the genetic expectation of a QTL effect obtained is the additive effect (A) when estimated from the RILs and RIL′s, the additive and dominance effects (A+D) from the BCF_1 mean values

Var%, Phenotypic variation explained by a single QTL
