# Supplementary material for: Main Effect QTL with Dominance Determines Heterosis for Dynamic Plant Height in Upland Cotton
Source: G3 (Bethesda). 2016 Aug 26;6(10):3373–9. doi: 10.1534/g3.116.034355 (PMC5068956; doi:10.1534/g3.116.034355)
Supplement: Supplemental Material [file supp_g3.116.034355_TableS4.pdf]

Table S4 Main effects and environmental interactions detected for plant height in RIL and RILV populations by inclusive composite interval mapping

Stage	Chr.	Position (cM)	Flanking markers		LOD	V(A)	V(AE)	A	AE1	AE2
RIL population										
*t*1	2	14	SWU12025	SWU11889	2.62	1.89	0.69	-0.51	-0.31	0.31
	5	115	TMB1296	HAU1603	5.62	4.52	3.34	0.78	0.67	-0.67
	10	42	SWU20260	Gh144	2.97	2.04	0.10	-0.52	-0.12	0.12
	11	95	CGR5421	ICR08245	2.72	0.03	1.72	0.07	0.48	-0.48
	11	201	NAU1014	ICR10344	3.25	1.62	0.02	0.47	-0.06	0.06
	16	79	C2_0011B	DC20124	2.90	1.57	0.01	0.46	-0.03	0.03
	19	6	NAU5330	Gh72	26.61	18.08	0.06	1.57	-0.09	0.09
	20	132	SWU20035	DPL0319	4.75	2.96	3.22	0.63	0.66	-0.66
	31	36	SWU16753	SWU16780	3.90	1.19	0.38	-0.40	0.23	-0.23
*t*2	10	42	SWU20260	Gh144	3.34	2.53	0.09	-0.90	-0.17	0.17
	11	201	NAU1014	ICR10344	3.88	2.64	0.00	0.92	-0.01	0.01
	19	6	NAU5330	Gh72	24.88	20.95	0.01	2.60	0.04	-0.04
	20	132	SWU20035	DPL0319	4.05	2.27	2.53	0.85	0.90	-0.90
*t*3	10	42	SWU20260	Gh144	5.11	4.33	0.02	-1.51	0.10	-0.10
	11	201	NAU1014	ICR10344	4.53	4.00	0.13	1.45	0.26	-0.26
	19	6	NAU5330	Gh72	14.15	13.13	0.20	2.64	-0.32	0.32
	20	129	SWU20035	DPL0319	2.99	1.51	1.30	0.89	0.82	-0.82
	24	3	Gh298	SWU13133	2.91	2.48	0.12	-1.14	-0.26	0.26
	25	131	Gh220	SWU19434	2.92	2.39	0.29	1.12	0.39	-0.39
*t*4	5	115	TMB1296	HAU1603	3.09	1.26	1.34	0.90	-0.93	0.93
	10	42	SWU20260	Gh144	5.70	4.90	0.20	-1.77	0.36	-0.36
	11	201	NAU1014	ICR10344	5.90	5.42	0.04	1.86	-0.16	0.16
	19	7	Gh616	CIR139	6.02	5.51	0.09	1.90	-0.24	0.24
	20	129	SWU20035	DPL0319	3.34	1.71	1.85	1.04	1.09	-1.09
	25	128	SWU19144	Gh220	2.75	2.67	0.01	1.30	0.09	-0.09
*t*5	4	0	SWU18881	NAU2701	2.55	2.62	0.00	1.42	0.05	-0.05
	10	42	SWU20260	Gh144	3.53	3.47	0.01	-1.64	0.07	-0.07
	11	201	NAU1014	ICR10344	6.93	7.20	0.08	2.35	-0.25	0.25
	19	9	Gh616	CIR139	2.92	3.00	0.02	1.56	-0.12	0.12
	24	67	BNL1521	HAU2504	3.07	2.25	0.80	1.31	-0.78	0.78
	25	126	SWU19144	Gh220	3.61	3.67	0.02	1.68	0.12	-0.12
RILV population										
*t*1	1	183	HAU2489	DPL0790	4.65	3.23	1.60	0.46	0.33	-0.33
	14	1	HAU0883	CIR228	4.05	3.43	0.25	0.47	0.13	-0.13

Stage	Chr.	Position (cM)	Flanking markers		LOD	V(A)	V(AE)	A	AE1	AE2
	16	61	C2_0011B	SWU10211	3.39	2.33	0.21	0.39	-0.12	0.12
	19	18	HAU3069	SWU17789	3.16	2.58	0.05	0.41	0.06	-0.06
	23	0	CGR5158	HAU1758	5.57	1.66	4.12	-0.34	-0.54	0.54
	23	202	DC40286	PGML1434	14.29	0.20	12.34	-0.12	-0.90	0.90
	29	12	SHIN0830	Gh111	3.62	3.07	0.26	0.45	0.13	-0.13
	30	18	TMB1638	CGR6812	3.52	2.25	0.33	-0.38	0.15	-0.15
*t*2	13	33	NAU3398	CGR5331	3.16	2.44	0.87	-0.61	-0.36	0.36
	23	0	CGR5158	HAU1758	6.48	1.67	5.50	-0.52	-0.94	0.94
	23	202	DC40286	PGML1434	7.89	1.47	6.81	-0.47	-1.02	1.02
	32	2	TMB0071	HAU1000	3.29	2.89	0.38	-0.66	0.24	-0.24
*t*3	13	33	NAU3398	CGR5331	4.16	2.78	1.36	-1.04	-0.72	0.72
	23	0	CGR5158	HAU1758	8.87	3.46	6.09	-1.20	-1.59	1.59
	23	200	NAU2140	DC40286	8.42	0.58	7.69	-0.48	-1.73	1.73
	29	12	SHIN0830	Gh111	2.82	2.56	0.17	1.00	-0.26	0.26
	32	4	HAU1000	TMB1931	3.06	2.95	0.01	-1.07	0.05	-0.05
*t*4	13	17	SWU13032	HAU2850	3.35	1.98	0.98	-0.98	0.69	-0.69
	13	33	NAU3398	CGR5331	5.68	3.52	1.46	-1.30	-0.84	0.84
	16	61	C2_0011B	SWU10211	3.89	2.56	1.14	1.11	-0.74	0.74
	23	0	CGR5158	HAU1758	8.03	2.62	4.73	-1.16	-1.56	1.56
	23	202	DC40286	PGML1434	9.37	3.37	5.10	-1.28	-1.57	1.57
	32	3	TMB0071	HAU1000	4.00	3.16	0.57	-1.23	0.52	-0.52
*t*5	13	33	NAU3398	CGR5331	6.50	5.29	0.37	-1.68	-0.45	0.45
	16	61	C2_0011B	SWU10211	3.10	2.69	0.71	1.20	-0.62	0.62
	23	0	CGR5158	HAU1758	8.44	2.17	5.08	-1.12	-1.71	1.71
	23	61	SHIN1076	BNL3482	2.54	1.83	0.23	1.02	0.36	-0.36
	23	202	DC40286	PGML1434	11.48	2.74	7.21	-1.22	-1.97	1.97
	29	12	SHIN0830	Gh111	2.70	2.31	0.43	1.11	-0.48	0.48

Chr represents the linkage group number of the loci being tested in the analysis.

V(A): Phenotypic variation explained by additive effect at the current scanning position.

V(AE): Phenotypic variation explained by additive by environment effect at the current scanning position.

A, the additive effect.
