# Supplementary material for: Main Effect QTL with Dominance Determines Heterosis for Dynamic Plant Height in Upland Cotton
Source: G3 (Bethesda). 2016 Aug 26;6(10):3373–9. doi: 10.1534/g3.116.034355 (PMC5068956; doi:10.1534/g3.116.034355)
Supplement: Supplemental Material [file supp_g3.116.034355_TableS5.pdf]

Table S5 Main effects and environmental interactions detected for plant height in BCF_1 and $BCVF_1$ populations by inclusive composite interval mapping

Trait	Chr.	Position (cM)	Flanking markers		LOD	V(A)	V(AE)	A	AE1	AE2
BCF_1 population										
*t*1	7	68	CGR5372	SWU10205	5.86	4.84	0.29	-0.60	-0.15	0.15
	16	79	C2_0011B	DC20124	4.56	2.38	0.52	0.42	-0.20	0.20
	19	7	Gh616	CIR139	22.56	18.75	0.22	1.20	-0.13	0.13
	20	26	CGR6154	SWU20246	2.80	1.90	0.69	-0.38	-0.23	0.23
	21	252	DPL0777	CGR5217	3.34	1.28	2.13	0.31	0.40	-0.40
	26	51	BNL2495	DPL0491	2.94	2.08	0.75	-0.40	-0.24	0.24
*t*2	4	2	SWU18881	NAU2701	3.12	2.33	0.09	0.62	-0.12	0.12
	7	66	CGR5372	SWU10205	2.94	2.26	0.00	-0.61	0.01	-0.01
	19	7	Gh616	CIR139	22.12	21.45	0.07	1.91	0.11	-0.11
	20	90	CER0167	SWU20064	5.84	4.55	0.00	-0.87	0.00	0.00
	21	254	DPL0777	CGR5217	2.94	1.63	1.52	0.52	0.50	-0.50
	26	82	CGR6930	SWU17241	5.06	4.45	0.39	-0.86	-0.25	0.25
*t*3	4	3	NAU2701	SWU18876	3.14	3.16	0.13	0.99	-0.20	0.20
	7	67	CGR5372	SWU10205	2.82	2.76	0.29	-0.92	0.30	-0.30
	19	7	Gh616	CIR139	7.60	3.52	3.16	1.05	1.00	-1.00
	20	63	SWU20246	SWU20501a	2.54	2.45	0.00	-0.87	0.01	-0.01
	20	90	CER0167	SWU20064	3.57	3.29	0.01	-1.01	0.04	-0.04
	21	254	DPL0777	CGR5217	2.98	0.76	1.77	0.48	0.74	-0.74
	26	82	CGR6930	SWU17241	5.27	5.23	0.01	-1.27	-0.05	0.05
*t*4	4	2	SWU18881	NAU2701	4.50	5.06	0.68	1.36	-0.50	0.50
	5	155	SWU13378	SWU17846	2.71	2.39	0.00	0.94	0.02	-0.02
	19	6	NAU5330	Gh72	4.98	4.02	0.09	1.22	0.18	-0.18
	21	254	DPL0777	CGR5217	3.96	0.72	1.91	0.51	0.83	-0.83
	25	0	HAU1382	SWU19848	2.50	0.58	2.84	0.46	-1.02	1.02
*t*5	4	3	NAU2701	SWU18876	4.75	5.28	0.77	1.49	-0.57	0.57
	5	155	SWU13378	SWU17846	4.38	3.44	0.02	1.21	0.10	-0.10
	14	58	NAU3308	HAU1057	2.51	0.06	1.60	-0.16	-0.82	0.82
	20	25	CGR6154	SWU20246	2.97	0.54	1.09	-0.48	-0.68	0.68
	21	252	DPL0777	CGR5217	3.83	0.69	1.52	0.54	0.80	-0.80
	25	1	HAU1382	SWU19848	2.76	0.27	2.82	0.34	-1.09	1.09
$BCVF_1$ population										
*t*1	4	58	SWU16783	SWU18876	4.18	4.10	1.17	0.40	0.21	-0.21
	23	204	DC40286	PGML1434	4.36	0.15	4.42	-0.08	-0.42	0.42
*t*2	13	23	SWU13032	HAU2850	2.53	2.65	0.04	-0.49	-0.06	0.06

Trait	Chr.	Position (cM)	Flanking markers		LOD	V(A)	V(AE)	A	AE1	AE2
	15	11	NAU3736	SWU11691	3.33	3.44	0.01	0.56	-0.03	0.03
	23	202	DC40286	PGML1434	8.16	0.64	8.99	-0.25	-0.92	0.92
*t*3	13	25	SWU13032	HAU2850	2.92	3.23	0.00	-0.75	0.01	-0.01
	15	11	NAU3736	SWU11691	2.59	2.97	0.02	0.72	-0.06	0.06
	23	201	DC40286	PGML1434	3.42	0.35	4.02	-0.25	-0.84	0.84
*t*4	21	108	PGML2500	CGR6521	2.96	3.01	0.09	-0.81	-0.14	0.14
	23	202	DC40286	PGML1434	3.37	0.12	3.27	-0.16	-0.84	0.84
	26	28	CGR6477	PGML2562	4.24	3.70	0.54	-0.90	0.34	-0.34
	28	1	BNL3545	PGML3983	3.55	2.11	1.48	-0.67	0.56	-0.56
	37	38	JESPR154	BNL5602	3.21	1.29	2.33	-0.53	-0.71	0.71
*t*5	13	29	SWU13032	HAU2850	3.11	2.95	0.01	-0.91	0.05	-0.05
	23	204	DC40286	PGML1434	3.39	1.49	2.31	-0.65	-0.81	0.81
	26	27	CGR6477	PGML2562	3.80	3.63	0.33	-1.02	0.31	-0.31
	28	1	BNL3545	PGML3983	2.51	2.18	0.30	-0.78	0.29	-0.29
	31	123	SWU16721	SWU16680	2.64	2.08	0.40	0.77	-0.34	0.34
	34	5	JESPR297	ICR00647	3.30	2.28	1.61	0.80	0.67	-0.67
	37	36	JESPR154	BNL5602	3.90	1.34	3.16	-0.61	-0.94	0.94

See footnotes of supplementary table S1 for explanations
