# Supplementary material for: Main Effect QTL with Dominance Determines Heterosis for Dynamic Plant Height in Upland Cotton
Source: G3 (Bethesda). 2016 Aug 26;6(10):3373–9. doi: 10.1534/g3.116.034355 (PMC5068956; doi:10.1534/g3.116.034355)
Supplement: Supplemental Material [file supp_g3.116.034355_TableS6.pdf]

Table S6 Epistatic effects and environmental interactions detected for plant height in RIL and RILV populations using two-locus analysis by inclusive composite interval mapping

Stage	Chi	Flanking markers		Chj	Flanking markers		LOD	V(AA)	V(AAE)	AA	AAE1	AAE2
RIL population												
*t*1	6	ICR00143	CGR5108	9	SWU15511	SWU15413	5.33	2.72	1.07	0.61	0.38	-0.38
	9	Gh111	Gh27	14	ICR12037	CGR5675	5.92	3.12	1.39	-0.65	-0.43	0.43
	14	BNL3661	PGML249	19	NAU5330	Gh72	5.40	3.03	1.43	-0.64	-0.44	0.44
	7	PGML1916	SWU1086	19	SWU17782	DPL0056	5.83	1.85	1.97	0.55	0.56	-0.56
	9	HAU1618	NAU2873	22	SWU21635	SWU21646	5.15	2.77	1.05	0.62	0.38	-0.38
	14	ICR12037	CGR5675	22	SWU21646	SWU21585	5.59	2.24	1.62	0.55	0.47	-0.47
	18	Gh60	SWU2228	22	SWU21533	DPL0562	5.05	1.40	2.38	0.44	0.57	-0.57
	18	DC40150	ICR02849	24	SWU13267	BNL1521	5.29	1.61	1.66	-0.50	-0.50	0.50
	9	Gh158	DC40407	26	BNL598	PGML163	5.97	1.62	2.93	-0.47	-0.63	0.63
	29	DC20127	DPL0252	32	NAU2140	NAU2957	5.81	2.22	2.14	0.56	0.54	-0.54
*t*2	14	ICR12037	CGR5675	22	SWU21646	SWU21585	5.54	2.39	1.51	0.87	0.69	-0.69
	18	Gh60	SWU2228	22	SWU21533	DPL0562	5.02	1.67	2.05	0.75	0.82	-0.82
	14	TMB0071	BNL3661	26	SWU18681	SWU0598	5.61	2.87	1.19	0.96	0.62	-0.62
	21	HAU0423	CGR5806	27	SWU11384	ICR11885	6.24	2.71	1.55	-0.96	-0.72	0.72
	29	DPL0252	BNL3261	32	NAU2140	NAU2957	5.16	1.32	2.32	0.66	0.87	-0.87
*t*3	13	DPL0535	CER0165	25	SWU19129	PGML285	6.02	2.42	2.47	-1.08	-1.17	1.17
	14	TMB0071	BNL3661	26	SWU18681	SWU0598	5.41	3.00	1.04	1.25	0.74	-0.74
*t*4	1	NAU6367	MUSS422	10	NAU2139	SWU20689	5.33	1.09	3.42	-0.84	-1.51	1.51
	1	SWU0077	HAU1417	11	NAU3695	DPL0050b	7.38	4.74	1.69	-1.76	-1.05	1.05
	16	SWU10627	PGML130	30	BNL243	CER0168	5.51	1.97	2.95	1.13	1.38	-1.38
*t*5	23	SWU14807	PGML418	25	BNL3594	DPL0282	5.03	3.43	0.96	-1.64	0.88	-0.88
RILV population												
*t*1	1	ICR03724	SWU1163	5	Gh260	PGML012	5.20	1.84	1.76	0.36	0.35	-0.35
	16	HAU3081	NAU747	23	DC40286	PGML143	5.66	3.86	0.34	0.50	-0.13	0.13
*t*2	1	SWU11632	SWU2195	11	CGR5421	MUSS278	5.63	4.97	0.08	0.87	0.13	-0.13
*t*5	2	DPL0200	SWU1188	16	HAU1129	C2_0011B	5.15	4.40	0.10	1.54	-0.23	0.23
	13	NAU3398	CGR5331	30	TMB1638	CGR6812	5.34	4.19	0.03	-1.50	-0.13	0.13

Chi and Chj represent the linkage group number of the loci being tested in the analysis

AA is the epistatic effect between loci i and j

AAE is the effect of the environmental interaction of epistasis

V(AA)% and V(AAE)%, percentage of the total variation explained by the AA and AAE
