# Supplementary material for: Main Effect QTL with Dominance Determines Heterosis for Dynamic Plant Height in Upland Cotton
Source: G3 (Bethesda). 2016 Aug 26;6(10):3373–9. doi: 10.1534/g3.116.034355 (PMC5068956; doi:10.1534/g3.116.034355)
Supplement: Supplemental Material [file supp_g3.116.034355_TableS7.pdf]

Table S7 Epistatic effects and environmental interactions detected for plant height in BCF_1 and $BCVF_1$ populations using two-locus analysis by inclusive composite interval mapping

Stage	Chi	Flanking markers		Chj	Flanking markers		LOD	V(AA)	V(AAE)	AA	AAE1	AAE2
BCF_1 population												
*t*1	3	SWU12783	SWU1281	3	SWU12765	NAU3839	5.38	3.61	0.24	0.58	0.12	-0.12
	13	SWU22309	SWU2232	24	BNL1521	HAU2504	5.08	2.94	0.84	-0.48	-0.26	0.26
	6	HAU1460	HAU1371	26	BNL598	PGML163	5.04	1.79	1.89	-0.37	-0.38	0.38
*t*3	5	HAU1603	PGML445	9	CGR6876	CGR5758	5.01	3.59	1.17	-1.05	0.62	-0.62
	6	ICR10602	SWU1965	30	CER0168	SWU21718	5.08	2.44	2.15	-0.86	0.82	-0.82
*t*4	1	BNL2827a	NAU6367	2	SWU11976	SWU11950	5.03	2.26	2.46	0.92	-0.96	0.96
	11	CAU0003	DC40250	13	DPL0535	CER0165	5.04	2.71	2.07	1.00	-0.88	0.88
	4	SWU16783	NAU3868	14	PGML4763	SWU13909	5.72	3.12	2.66	1.07	-0.99	0.99
	11	CAU0003	DC40250	18	DC40150	ICR02849	5.34	2.89	2.23	1.04	-0.92	0.92
	8	HAU3177	NAU4064	26	DPL0491	Gh64	5.02	3.05	1.98	-1.05	0.85	-0.85
	7	SWU10064	NAU3181	26	SWU18681	SWU0598	6.71	5.07	1.46	-1.36	0.73	-0.73
	16	SWU10266	DC40065	32	NAU2140	NAU2957	5.11	3.55	1.57	1.14	-0.76	0.76
*t*5	4	SWU16782	SWU1678	7	CGR5001	CGR6586	5.34	4.07	1.41	1.30	-0.78	0.78
	9	HAU190	HAU1618	23	PGML4186	NAU3100	5.53	1.96	4.07	0.91	-1.31	1.31
	12	NAU943	DPL0303	25	BNL3594	DPL0282	5.49	3.79	1.80	-1.28	0.88	-0.88
	7	SWU10064	NAU3181	26	SWU18681	SWU0598	6.83	5.29	1.45	-1.49	0.78	-0.78
	10	SWU20501	CGR5873	29	DC20127	DPL0252	5.10	2.68	2.13	-1.08	0.95	-0.95
$BCVF_1$ population												
*t*3	6	BNL3650	TMB2940	23	CGR5158	HAU1758	5.55	5.19	0.43	0.99	0.28	-0.28
	23	CGR5158	HAU1758	23	NAU2140	DC40286	6.32	5.77	0.31	1.02	0.15	-0.15
	20	SWU20027	Gh187	24	HAU3076	SWU13121	5.05	5.29	0.01	-0.96	-0.07	0.07
*t*4	17	HAU1413	CGR5576	26	MGHES31	HAU1571	5.13	3.71	0.92	0.91	0.46	-0.46
	31	CGR6772	HAU0355	31	SWU16780	SWU16735	5.26	3.45	1.03	0.91	0.56	-0.56
*t*5	36	CGR5548	SWU2070	38	NAU2450	PGML194	5.19	5.09	0.00	-1.22	0.05	-0.05

See footnotes of supplementary table S3 for explanations
